# No single best technique: trade-offs in femoral interference screw fixation for bone–patellar tendon–bone anterior cruciate ligament reconstruction

**DOI:** 10.1530/EOR-2025-0247

**Published:** 2026-07-01

**Authors:** Seikai Toyooka, Yutoshi Osaki, Noriaki Arai, Hirotaka Kawano, Takumi Nakagawa

**Affiliations:** Department of Orthopaedic Surgery, Teikyo University School of Medicine, Tokyo, Japan

**Keywords:** anterior cruciate ligament, bone–patellar tendon–bone, interference screw, knee joint

## Abstract

- This review presents how surgeons should choose femoral interference screw in anterior cruciate ligament (ACL) reconstruction – insertion direction (inside-out vs outside-in), tunnel shape (round vs rectangular), screw position (anterior vs posterior), and screw material (metal, bioabsorbable, PEEK, magnesium) – to balance fixation strength, anatomic fidelity, and revision feasibility.
- Inside-out maximizes aperture compression and preserves the lateral femoral cortex but requires deep flexion and precise trajectory (risk of iatrogenic cartilage contact/posterior wall breach). Outside-in improves visualization and guide control yet needs a large lateral cortical socket and can push the bone plug intra-articularly as the screw advances from cortex to joint.
- Rectangular tunnels better match the native footprint, improving rotational control and interface congruity, while round tunnels simplify preparation but allow plug rotation and may use bone less efficiently at the aperture.
- Posterior screw placement (particularly with the inside-out technique) enhances cancellous-to-cancellous compression but risks posterior cortical breach injury; anterior placement protects fibers and favors footprint fidelity but may reduce cancellous compression.
- Metal offers high stiffness and no tapping but creates MRI artifact and requires removal if present at revision. Bioabsorbable materials avoid artifact/removal yet are weaker and may fracture in sclerotic tunnels. PEEK (metal-like without artifact) and magnesium (osteoconductive/resorbable) are promising but lack long-term data.
- No single method is universally optimal. Decisions should be individualized to visualization needs, cortical/bone-stock preservation, expected revision strategy, and patient factors (age, activity, bone quality).

## Introduction

Anterior cruciate ligament (ACL) reconstruction is one of the most frequently performed procedures in sports orthopedics. Among available graft options, the bone–patellar tendon–bone (BTB) autograft has remained a widely used benchmark owing to its superior initial fixation strength and the biological advantage of direct bone-to-bone healing (1, 2). Interference screw (IFS) fixation has become the predominant method for securing BTB grafts, offering aperture fixation and early stability (3, 4). Despite its widespread use, several controversies persist. First, surgeons differ in their preference for insertion technique – inside-out versus outside-in – each associated with distinct biomechanical and technical implications. Second, the optimal tunnel shape – round versus rectangular (noncircular) – is debated with respect to anatomic fidelity, rotational control, and bone preservation. Third, the ideal screw position relative to the bone plug – anterior versus posterior – and screw diameter relative to tunnel and plug remains uncertain. Finally, the choice of screw material – metallic versus bioabsorbable, with newer alternatives such as PEEK or magnesium – continues to be discussed.

Importantly, these issues are particularly relevant to femoral fixation, which often poses greater technical and mechanical challenges than tibial fixation in clinical practice (5, 6). Successful ACL reconstruction requires securing the femoral graft in an anatomically accurate position, yet it is also the site most commonly associated with complications such as tunnel malposition, cortical breach, insufficient fixation strength, tunnel enlargement, or difficulty during revision (7, 8). Therefore, understanding the nuances of femoral-side IFS fixation – its insertion methods, tunnel geometry, positional strategies, and material properties – is essential for optimizing outcomes in BTB ACL reconstruction. This article presents a narrative review of the available literature focusing specifically on femoral interference screw fixation in BTB ACL reconstruction. Relevant biomechanical, clinical, and technical studies were synthesized to highlight current concepts, controversies, and practical implications for both primary and revision surgery.

### Insertion techniques

The insertion technique represents a key variable in BTB ACL reconstruction. The inside-out method involves screw placement from the joint surface outward, compressing the bone plug at the tunnel aperture. This provides strong aperture fixation and minimizes graft micromotion, thereby reducing tunnel widening (9). A distinct advantage is that the femoral socket can be prepared without breaching the lateral femoral cortex, preserving cortical bone stock for potential future revision (10). In practice, the inside-out method is most commonly performed through a far-medial portal. However, this approach requires deep knee flexion, often exceeding 120°, which not only restricts visualization but also increases the technical difficulty of advancing a screw or tap into the femoral tunnel (11, 12). In such situations, instruments may inadvertently contact and damage the articular cartilage of the medial femoral condyle (13, 14). Furthermore, accurate screw trajectory is demanding; if the tunnel is directed excessively posteriorly, the femoral posterior wall is at risk of cortical breach (‘blowout’), and fixation strength is also compromised when screw–graft divergence exceeds 15° (12).

By contrast, the outside-in method introduces the screw from the lateral cortex toward the joint. This technique facilitates precise anatomic tunnel placement and does not require extreme flexion, thereby offering improved visualization and surgical control (15, 16). However, outside-in requires a relatively large lateral cortical socket (17). While this seldom affects primary outcomes, the cortical violation reduces bone stock and the intact rim, thereby complicating treatment, especially when multiple ligament injuries require numerous bone tunnels. In addition, because the screw is driven from the lateral cortex toward the joint, the screw tip can push the femoral bone plug into the joint space before it fully engages (‘graft push-in’) (18, 19).

**Figure 1:**
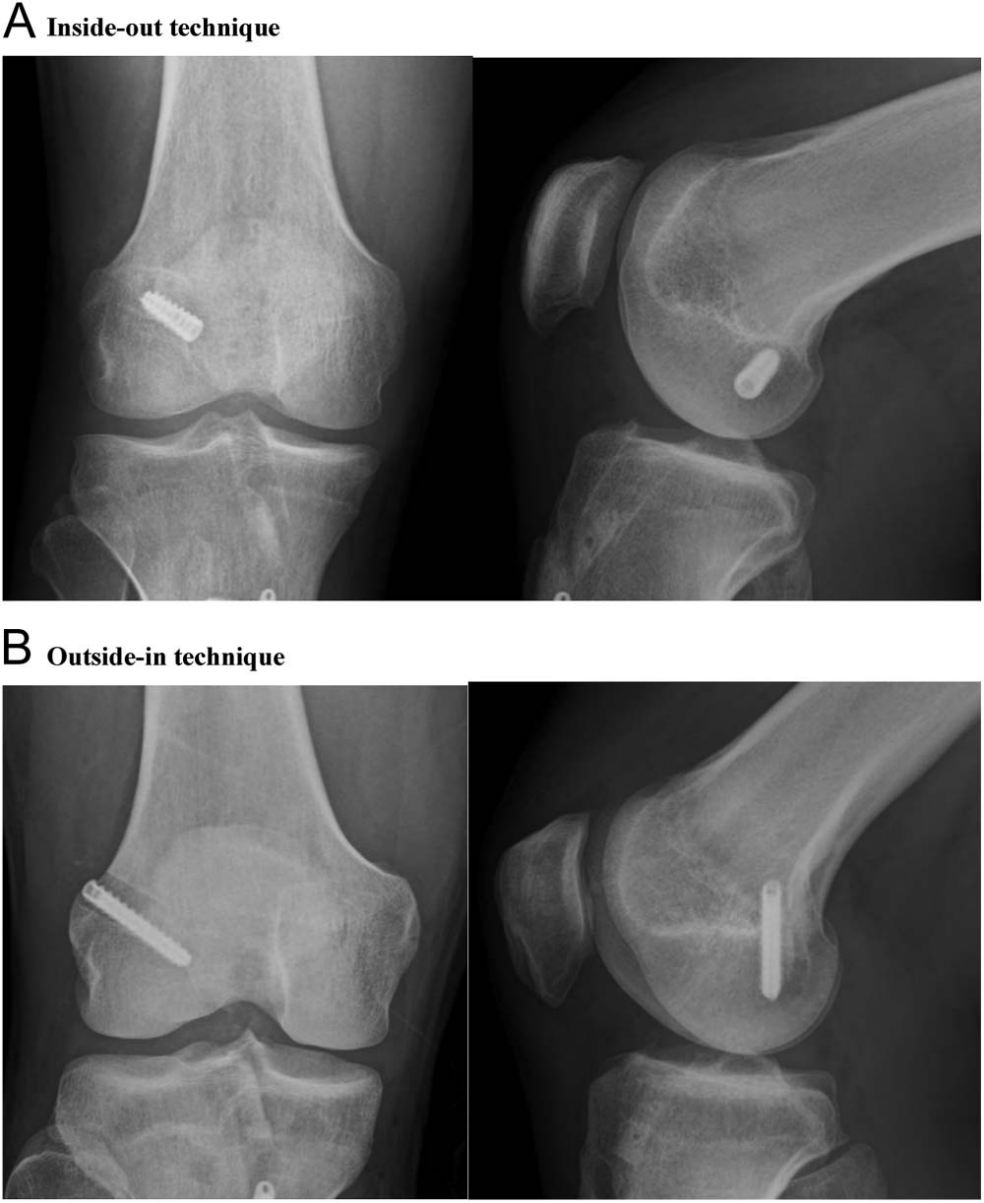
Plain X-ray with BTB fixed by metal interference screw: (A) inside-out technique and (B) outside-in technique.

Biomechanical studies generally support the superiority of aperture fixation, and the reduced graft bending angle is an advantage of the inside-out approach (20, 21, 22). However, the outside-in technique offers the advantages of longer bone tunnel length, and short-term clinical outcomes remain comparable (9, 21, 23). Thus, the choice between inside-out and outside-in should not be based solely on primary fixation strength but must incorporate considerations of technical feasibility, intraoperative visualization, the risk of iatrogenic cartilage injury, and – most importantly – the preservation of cortical bone stock and fixation options in the event of revision surgery. Figure 1 shows the simple X-ray for both techniques, and Table 1 shows their pros and cons.

**Table 1:** Comparison of inside-out vs outside-in interference screw insertion in BTB ACL reconstruction: pros and cons.

Aspect	Inside-out (transportal)	Outside-in
Visualization/working angle	Requires deep flexion (>120°); narrower view; harder instrument maneuvering	No extreme flexion; improved visualization and guide control
Aperture fixation	Strong aperture compression; less tunnel widening	Fixation tends to be farther from the aperture
Lateral femoral cortex	Preserved: no lateral cortical socket	Requires a large lateral cortical socket: bone stock reduced
Graft-bending angle	Generally smaller	Generally more acute (greater shear concern)
Femoral tunnel length	Typical/standard	Often longer (greater graft–tunnel contact length)
Cartilage injury risk	Risk of iatrogenic contact with medial femoral condyle cartilage in deep flexion	Lower risk (less flexion required)
Posterior wall breach	Higher risk if trajectory drifts posteriorly (posterior cortical ‘blowout’)	Typically, easier to avoid with controlled guide trajectory
Graft push-in (plug advancement)	Uncommon	Screw advances from cortex toward joint: may push bone plug intra-articularly if unmitigated
Instrumentation	Standard cannulated reamers; no lateral socket instrumentation	Often uses outside-in systems; additional instrumentation needed

### Tunnel shape: round vs rectangular in femoral BTB fixation

Noncircular (rectangular or ovalized) femoral tunnels were introduced to more faithfully reproduce the ribbon-like morphology of the native ACL femoral footprint (24, 25). In the BTB setting, this geometry aligns the flat cortical face of the bone plug with the noncircular tunnel walls, improving rotational control at the aperture and potentially enhancing anatomic footprint coverage (26, 27). A practical corollary is increased interface congruity: because the rectangular socket more closely matches the plug’s cross section, contact area per unit circumference is greater and toggle is reduced. This can improve aperture compression and early graft stability, particularly when the screw is positioned to augment cancellous-to-cancellous contact (28). Clinically, many surgeons report that this geometry helps mitigate rotational drift of the bone plug during screw insertion, thereby preserving the intended posterior fiber orientation toward the native footprint (16, 29, 30).

These advantages, however, come with technical costs. Creating a rectangular tunnel is more labor-intensive than drilling a standard round tunnel and typically requires specialized guides or energy devices (e.g. ultrasonic chisels/reamers) designed for noncircular preparation. Precision is critical at the tunnel corners, where over-resection can blunt the geometry and under-resection can impede passage. Because the socket closely conforms to the plug, there is less ‘play’ during insertion; seating the plug becomes a more technical maneuver, often requiring meticulous trajectory control and controlled impaction to avoid chondral contact in deep flexion (inside-out) or posterior cortical encroachment when the screw trajectory tends posteriorly (26). The snug fit can also increase frictional resistance; surgeons should be prepared to modulate screw size, tapping strategy (for weaker materials), and insertion torque to prevent iatrogenic plug fragmentation (12).

By contrast, a round tunnel is fast, familiar, and equipment-agnostic. It can be created with standard cannulated reamers using either inside-out or outside-in drilling, and plug insertion is generally easier because the circular geometry provides a modest circumferential clearance that aids passage and seating. This simplicity and reproducibility are major reasons the round tunnel remains the default in many practices (31). The trade-off is that the mismatch between a flat-sided bone plug and a circular socket permits rotational slippage – particularly during screw insertion – so the plug may rotate anteriorly or posteriorly. While efforts to match the shape – such as using a triangular bone block – can mitigate this, they may slightly alter the intended fiber orientation or the fidelity of the point. Surgeons often counter this with intra-tunnel anti-rotation maneuvers (e.g. holding sutures under tension, provisional pins) or by choosing screw position (anterior vs posterior) to bias the plug against the desired wall (32).

Another practical distinction emerges when considering bone stock and future revision. Compared to circular bone tunnels, rectangular bone tunnels have been reported to have a smaller surface area. Rectangular tunnels have been proposed to reduce circumferential over-resection compared with round tunnels by better matching the footprint’s noncircular shape, thereby potentially preserving more cancellous bone for future revision strategies (30). Contemporary clinical series and reviews of noncircular tunnels generally show comparable short-term outcomes to round tunnels, while technique articles emphasize that footprint-conforming preparation can facilitate early plug–socket integration in BTB constructs (33, 34, 35).

**Figure 2:**
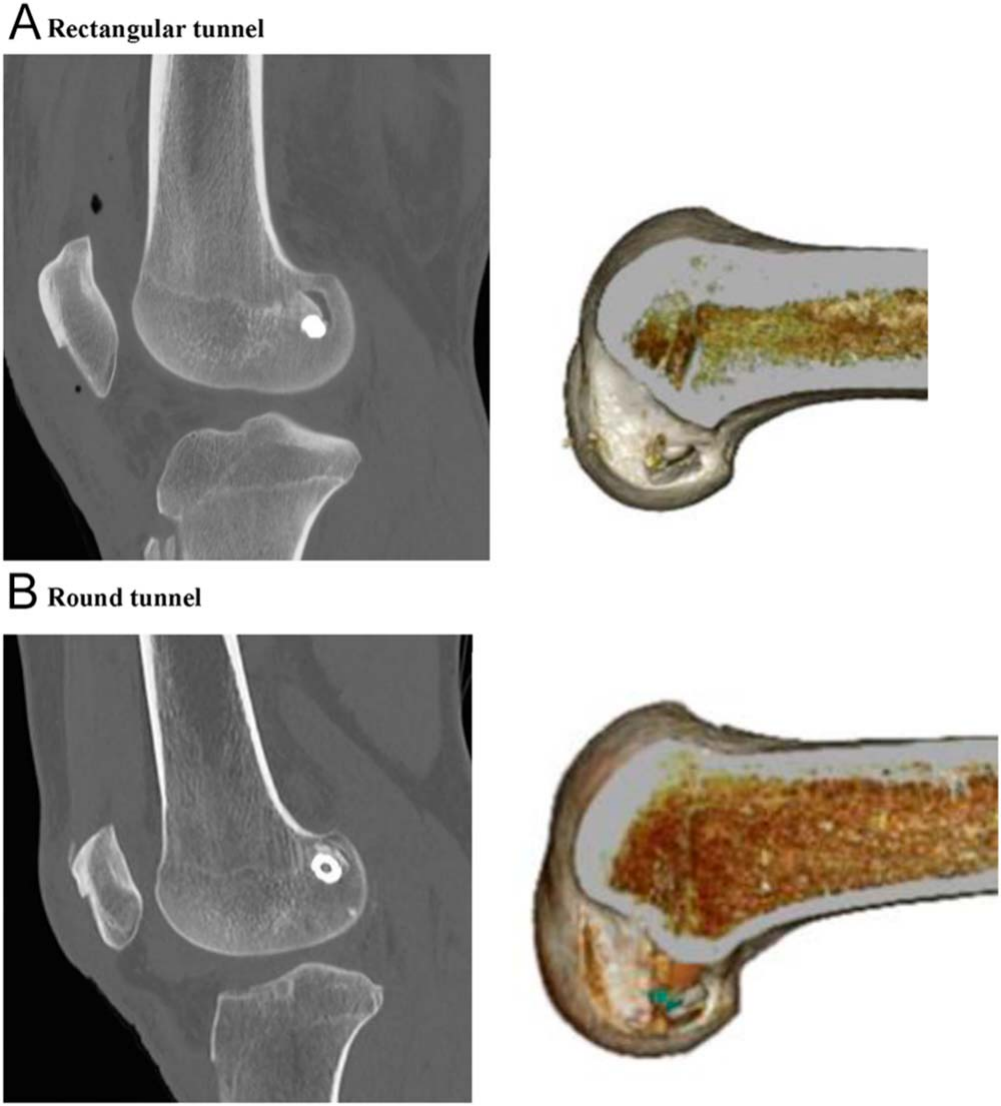
CT images with BTB fixed by interference screw: (A) rectangular tunnel and (B) round tunnel.

In summary, rectangular tunnels offer anatomic fidelity and rotational stability with potentially greater interface compression but demand specialized instruments and higher technical precision during preparation and plug insertion (26, 27). Round tunnels provide speed, simplicity, and easier seating at the expense of greater rotational susceptibility during screw insertion and less efficient use of bone at the aperture. Selection should be guided by the surgeon’s priorities – anatomic reproduction and rotational control versus workflow simplicity and device availability – while keeping a long-term perspective on bone preservation for revision (34). Figure 2 shows the CT images for both techniques, and Table 2 shows their pros and cons.

**Table 2:** Comparison of femoral tunnel shapes in BTB ACL reconstruction: pros and cons.

Aspect	Rectangular/noncircular tunnel	Round tunnel
Anatomic fidelity (footprint match)	Closer to ribbon-like native ACL footprint; better footprint coverage	Less anatomical; circular shape may over-/under-cover parts of footprint
Rotational stability of bone plug	High-flat walls resist plug rotation; helps preserve posterior fiber orientation during screw insertion	Lower-plug can rotate during screw insertion; risk of subtle fiber/footprint drift
Interface contact and aperture compression	Greater conforming contact; efficient aperture compression with posterior or anterior screw bias	Less conforming; compression less focused unless aided by screw positioning/anti-rotation maneuvers
Bone preservation at aperture (cross-sectional area)	Typically smaller tunnel area for comparable footprint coverage: more bone preserved	Generally, larger area removed for same coverage: less bone preserved
Revision friendliness	Favorable: more cancellous bone retained; facilitates re-tunneling/bone grafting options	Less favorable: larger aperture area can limit options; may require more grafting
Instrumentation and setup	Requires specialized guides/slot reamers/ultrasonic chisels; limited availability in some centers	Standard cannulated reamers; widely available; no special devices needed
Technical demand (learning curve)	High – precise corner preparation; tight tolerance increases sensitivity to trajectory and divergence	Low–moderate – familiar, reproducible, broad comfort among surgeons
Bone plug insertion	Technically demanding: minimal ‘play’, higher friction; controlled impaction/ trajectory needed	Easier: modest circumferential clearance aids passage and seating
Risk during screw insertion	Lower risk of plug rotation; but tight fit can increase insertion torque demands	Higher risk of plug rotation unless controlled (e.g. holding sutures, provisional pins, triangular bone plug)

### Screw positioning: anterior vs posterior to the bone plug

The precise positioning of the interference screw relative to the bone plug introduces further considerations. In standard practice, the femoral bone plug is typically inserted with its cancellous surface anterior and the tendon fibers posterior, reflecting the posterior location of the native ACL femoral footprint (26, 36). This orientation allows the graft to be subjected to anteriorly directed forces that compress the cancellous bone against the anterior tunnel wall, thereby promoting bone-to-bone healing while simultaneously reproducing the native fiber alignment (37, 38). At this stage, the surgeon must choose between posterior screw placement and anterior screw placement.

Posterior screw placement compresses the cancellous bone surface firmly against the anterior tunnel wall, maximizing bone-to-bone contact and promoting osseous integration (38, 39). However, placing the screw posterior to the graft carries several disadvantages. First, when the screw is directed posteriorly – especially in inside-out techniques – it is more likely to approach the posterior aspect of the lateral femoral condyle, with a higher risk of breaching or damaging the posterior cortical wall (39, 40). Such posterior protrusion is less common with outside-in approaches, but remains a technical risk. Second, because tendon fibers of the graft are positioned posteriorly, screw or tap insertion in this region increases the likelihood of damaging graft fibers or sutures used for fixation.

Anterior screw placement, by contrast, avoids interference with the posteriorly located graft fibers and reduces the risk of damaging them during screw or tap insertion (41). It also lessens the risk of posterior cortical breach in the lateral femoral condyle, as the screw trajectory remains more anterior. Furthermore, anterior screw placement pushes the graft posteriorly against the tunnel, allowing it to rest closer to its anatomical femoral footprint. However, one potential disadvantage is that anterior placement may reduce direct cancellous-to-cancellous bone contact, thereby possibly limiting the quality of osseous healing.

**Figure 3:**
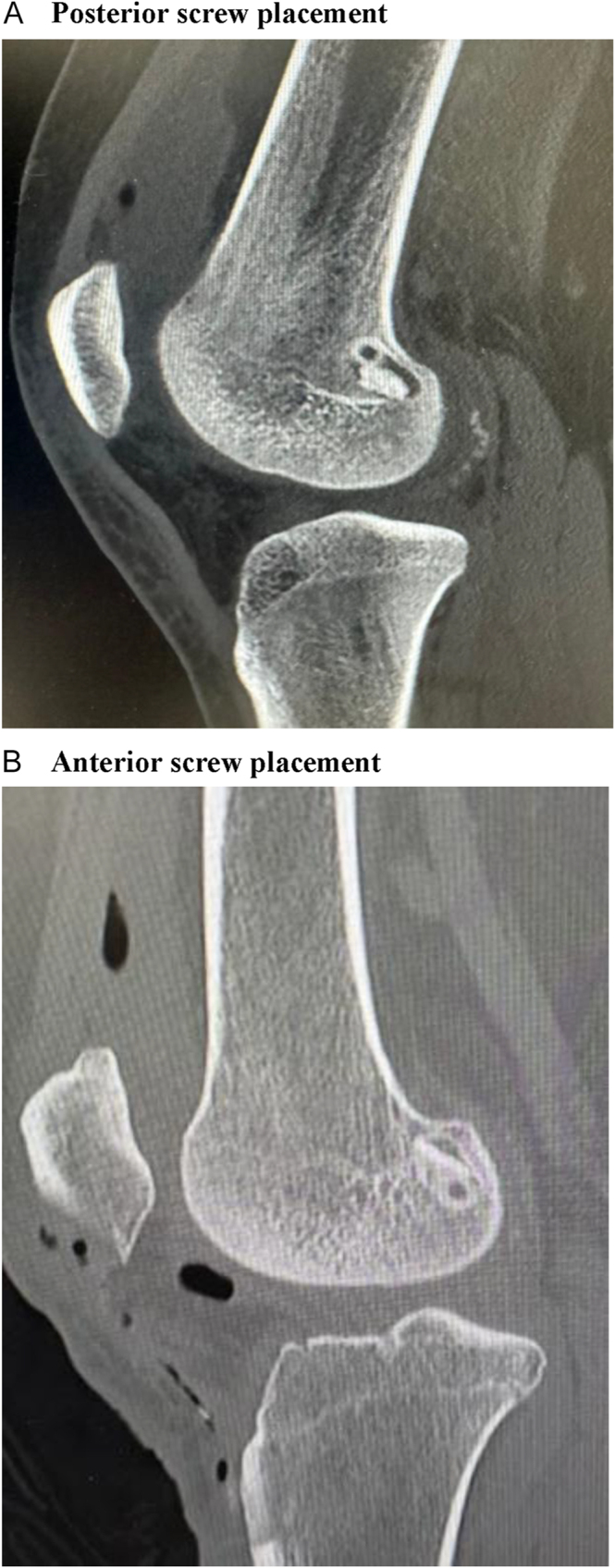
Sagittal view of CT showing BTB fixed with an absorbable interference screw: (A) posterior screw placement and (B) anterior screw placement.

Both strategies are viable, reflecting a balance between biological integration (maximized by posterior screw placement) and anatomical fidelity (preserved by anterior screw placement). To date, no randomized controlled trials or high-quality comparative studies have directly evaluated anterior versus posterior screw positioning in BTB femoral fixation. Limited evidence from soft tissue graft studies suggests that posterior screw placement may reduce tunnel widening, raising the hypothesis that anterior screw placement of the graft itself – by ensuring cancellous bone contact anteriorly – might prevent tunnel enlargement on the femoral side (42). However, these findings cannot be directly extrapolated. The absence of definitive data highlights a major gap in current knowledge and identifies an important area for future biomechanical and clinical investigation. Figure 3 shows the CT images for both techniques, and Table 3 shows their pros and cons.

**Table 3:** Comparison of anterior vs posterior screw placement in BTB ACL reconstruction: pros and cons.

Aspect	Anterior screw placement	Posterior screw placement
Relation to graft fibers	Avoids interference with posteriorly located tendon fibers; reduces risk of fiber	Screw or tap may injure posterior graft fibers
Graft orientation	Pushes graft posteriorly toward its anatomic femoral footprint	Maintains graft position anteriorly; may alter footprint fidelity
Bone-to-bone healing	Less cancellous-to-cancellous compression; potential limitation for osseous integration	Maximizes cancellous bone compression against anterior tunnel wall; enhances osseous integration
Risk to femoral condyle cortex	Lower risk of posterior cortical breach; screw trajectory remains more anterior	Higher risk of breaching/damaging posterior cortex, especially in inside-out insertion
Risk of graft push-out/protrusion	Lower	Higher if screw tip protrudes posteriorly

### Screw materials in BTB ACL reconstruction

Metallic interference screws, typically titanium or stainless steel, have long been regarded as the gold standard for BTB ACL fixation. Their advantages are well established: they provide strong mechanical fixation and high stiffness, with sharp threads that reliably engage bone plugs even in osteoporotic bone (8, 43). Owing to this strong engagement, metallic screws can be inserted without tapping, which not only simplifies the surgical procedure but also eliminates the risk of intraoperative screw breakage that can occur with bioabsorbable devices. Moreover, the absence of a tapping step avoids the potential hazard of inadvertently damaging graft fibers or cutting the sutures used for graft preparation and passage, thereby preserving graft integrity. These properties make metallic screw insertion straightforward and predictable, even in dense bone, and account for their consistent biomechanical superiority. Numerous studies confirm that metallic screws achieve the highest pullout strength and stiffness, and clinical outcomes have been excellent with durable long-term follow-up (8, 28, 43).

However, the very fact that metallic screws advance by tightly engaging bone and bone plugs can create unique challenges. As the screw progresses, stress may be propagated through the bone plug or tunnel, raising the possibility of posterior cortical breach if trajectory alignment is suboptimal. While this complication is uncommon when careful technique is used, it represents a disadvantage relatively unique to metallic screws compared to bioabsorbable devices. In addition, metallic screws produce MRI artifacts that limit postoperative imaging and must be removed in revision surgery (38). Screw removal is often technically difficult and can be associated with tunnel enlargement, cortical bone loss, and compromise of the graft bed (43).

Bioabsorbable screws were developed to circumvent these limitations. Composed of PLLA, PGA, or β-TCP composites, they are MRI-compatible, gradually resorb, and obviate the need for removal (44). Their theoretical advantage lies in simplifying revision procedures and promoting osseous integration. However, they are mechanically weaker than metal screws. Although its use is implant- and surgeon-dependent rather than systematic, tapping may be considered particularly in dense or sclerotic bone to reduce insertion torque and mitigate insertional fracture. A tapping step can increase technical demand and, if performed without protective measures, may pose a risk to graft fibers or sutures. In revision cases, where tunnels are frequently sclerotic, bioabsorbable screws are particularly prone to fracture, complicating rather than facilitating the procedure (45). Although the new-generation screws have been improved, bioabsorbable screws have been associated with cyst formation, tunnel widening, inflammatory reactions, and occasional migration (45, 46, 47). Large reviews and meta-analyses indicate that although long-term stability and functional outcomes are comparable to metallic screws, their complication profiles differ significantly (48, 49).

Emerging alternatives such as PEEK and magnesium screws attempt to combine the strengths of both categories. PEEK screws provide mechanical strength comparable to metal while avoiding MRI artifact, though they do not resorb (50, 51). Magnesium-based screws are bioresorbable and osteoconductive, and early studies have shown encouraging results (52, 53). Nevertheless, concerns about transient hydrogen gas release and the absence of long-term outcome data limit their current application. For now, these materials remain promising but incompletely validated options. Table 4 shows their pros and cons.

**Table 4:** Comparison of interference screw materials in BTB ACL reconstruction: pros and cons.

Feature	Metal screws	Bioabsorbable screws	PEEK/magnesium (emerging)
Mechanical strength	Very high; reliable fixation even in dense or osteoporotic bone	Lower; risk of breakage during insertion, especially in sclerotic bone	Comparable to metal (PEEK); promising but limited data for Mg
Thread characteristics/tapping	Sharp threads; no tapping required, simplifying insertion	Tapping may be used in dense/sclerotic bone; tapping step adds time and technical demand	PEEK similar to metal (no tapping); Mg may require careful technique
Effect on graft fibers/sutures	No tapping avoids risk of graft fiber or suture damage	Tapping can risk cutting graft fibers or sutures during preparation	No tapping (PEEK); minimal risk (Mg, data limited)
Bone/tunnel interaction	Strong engagement with bone plug; risk of posterior cortical breach if trajectory is suboptimal	Less aggressive engagement; lower risk of cortical breach but weaker fixation	Similar to metal (PEEK); Mg osteoconductive
Intraoperative breakage	Rare	Relatively common if not tapped properly	Low (PEEK); limited data (Mg)
Imaging	MRI artifact	MRI-compatible	MRI-compatible
Revision surgery	Removal required; may enlarge tunnels and cause cortical bone loss	No removal required, but screw breakage in sclerotic bone complicates revision	PEEK: no removal required; Mg resorbs, but long-term revision outcomes unclear
Complications	Rare hardware irritation; possible posterior cortex breach	Cyst formation, tunnel widening, inflammatory reaction, migration	Mg: transient hydrogen gas release; long-term outcomes lacking

## Discussion

This narrative review consolidates current concepts in femoral interference screw (IFS) fixation for bone–patellar tendon–bone (BTB) ACL reconstruction with the practical aim of clarifying trade-offs rather than prescribing a single superior technique. Although insertion direction, tunnel shape, screw position, and screw material are frequently framed as decisive levers, in reality, they are interdependent, case-specific, and sensitive to factors that extend beyond any simple decision rule. Patient age and activity, bone quality and morphology, graft geometry, visualization and access, concomitant procedures, and the surgeon’s technical proficiency and available instrumentation all interact to shape the safest and most durable plan. The appropriate role of this review is to articulate the practical advantages and liabilities of each option; the final selection remains a nuanced intraoperative judgment informed by those pros and cons (53, 54).

While no single rule applies, several common scenarios may suggest particular choices. For instance, in patients with obesity or limited knee flexion, one might reasonably favor outside-in over inside-out because it can offer better visualization and guide control without forcing extreme flexion. When rotational control is a priority – and where the surgeon’s skill set and team support permit – adopting a rectangular (noncircular) femoral socket may help limit plug rotation during screw insertion, acknowledging the greater technical demand. If the femoral tunnel lies somewhat anterior to the native footprint, an anterior screw may help reconstruct the anterior wall and bias the plug posteriorly toward the footprint. Finally, for younger athletes with a potentially higher lifetime risk of revision, some surgeons may consider bioabsorbable screws to avoid MRI artifact and hardware removal at reoperation. These examples are offered as plausible, context-dependent options rather than prescriptive rules, underscoring the need to align fixation choices with patient factors, bone quality, and anticipated revision strategy.

Revision surgery reveals the downstream consequences of index choices. The first step is a meticulous assessment of tunnel position and diameter and of the integrity of the lateral femoral cortex (54, 55). When prior tunnels are well positioned, only minimally widened, the original approach – including the same screw-positioning logic – can often be reused because local mechanics and fixation options remain favorable. In contrast, enlarged or malpositioned tunnels typically mandate a route different from the index trajectory; in such cases, plan an alternative approach or a staged strategy as needed. When widening is marked or bone stock is poor, consider staged bone grafting with delayed reconstruction; at re-tunneling, a noncircular, footprint-conforming socket may help conserve cancellous bone at the aperture.

Implant selection must be matched to sclerotic revision bone. In this setting, bioabsorbable screws are prone to intraoperative fracture and are best avoided; metallic or PEEK screws are generally more reliable, with tapping used selectively according to bone hardness and device requirements (8, 43). Regardless of implant, trajectory control is paramount: maintain minimal screw–graft divergence, and if work near the posterior wall is unavoidable, protect the cortex through guide adjustments and explicit contingency plans for potential blowout.

Screw position at revision should be adapted to the prevailing pattern of bone loss. Anterior deficiency favors anterior screw placement to reconstruct the anterior wall and simultaneously drive the plug posteriorly toward the native footprint. Throughout, the surgeon should be explicit about the aperture load path being created; fixation should be designed to engage the strongest remaining bone while restoring functional alignment of the graft within the femoral tunnel.

In sum, femoral IFS fixation in BTB ACL reconstruction is best approached as an exercise in informed, patient-specific trade-off management. The surgeon’s task is not to apply a universal rule, but to understand the strengths and liabilities of each technique and implant, align them with the case priorities and intraoperative realities, and preserve future options whenever possible. Decisions made at the index procedure – especially regarding lateral cortex integrity and cancellous bone at the aperture – shape the feasibility, safety, and success of whatever revision the patient may one day require.

### Limitations

This review has limitations. It is narrative in nature, subject to selection and publication bias, and not a systematic review. Available studies are heterogeneous, often examining hamstring or tibial grafts rather than BTB femoral fixation. Evidence directly comparing anterior versus posterior screw placement in BTB grafts is absent, limiting conclusions in this domain. Most clinical studies are short term and underpowered, leaving the long-term clinical significance of biomechanical findings uncertain. Finally, emerging materials such as PEEK and magnesium screws lack sufficient follow-up to justify definitive recommendations.

## Conclusion

Interference screw fixation remains the standard for BTB ACL reconstruction, yet no single approach is universally optimal. Each option – metallic or bioabsorbable screws, inside-out or outside-in insertion, and anterior or posterior positioning – offers distinct advantages and limitations. The key is not to seek one superior method, but to understand these trade-offs and tailor fixation strategy to the patient’s characteristics and the surgeon’s priorities. Informed, individualized decision-making is essential to optimize both primary outcomes and the feasibility of future revision surgery.

## ICMJE Statement of Interest

The authors declare that there is no conflict of interest that could be perceived as prejudicing the impartiality of the work reported.

## Funding Statement

This research received no specific grant from any funding agency in the public, commercial, or not-for-profit sectors.

## Author contribution statement

ST conceptualized this study and wrote the original draft. YO, NA, HK, and TN carried out data analysis and interpretation. All authors critically reviewed and revised the manuscript draft and approved the final version for submission.
